# scATAC-seq generates more accurate and complete regulatory maps than bulk ATAC-seq

**DOI:** 10.1038/s41598-025-87351-7

**Published:** 2025-01-29

**Authors:** E. Ravza Gur, Jim R. Hughes

**Affiliations:** 1 MRC WIMM Centre for Computational Biology, MRC Weatherall Institute of Molecular Medicine, Radcliffe Department of Medicine, University of Oxford, Oxford, OX3 9DS UK; 2 MRC Molecular Haematology Unit, Radcliffe Department of Medicine, MRC Weatherall Institute of Molecular Medicine, University of Oxford, Oxford, OX3 9DS UK

**Keywords:** Bulk ATAC-seq, scATAC-seq, Gene regulation, Regulatory elements, Chromatin accessibility, Clustering, Pseudo-bulking, Single-cell epigenomics, Computational biology and bioinformatics, Genetics

## Abstract

Bulk ATAC-seq assays have been used to map and profile the chromatin accessibility of regulatory elements such as enhancers, promoters, and insulators. This has provided great insight into the regulation of gene expression in many cell types in a variety of organisms. To date, ATAC-seq has most often been used to provide an average evaluation of chromatin accessibility in populations of cells. The development of a single cell approach (scATAC-seq) assay enables researchers to evaluate chromatin accessibility in individual cells and identify sub-groups in mixed populations of cells. To investigate the full potential of single-cell epigenomic data, we have comprehensively compared the information derived from bulk ATAC-seq and scATAC-seq in populations of cells. We found that the chromatin architecture signal is the same using bulk ATAC-seq and scATAC-seq to analyse aliquots of the same cell population. However, scATAC-seq provides substantially higher data quality compared to bulk ATAC-seq improving the sensitivity to detect relatively weak, but functionally important ATAC-seq signals. Furthermore, we found that scATAC-seq identified differences in what was previously assumed to be a homogenous population of cells. Finally, we determined the number of cells required to generate aggregated open chromatin profiles from single cells and to identify biologically meaningful clusters after pseudo-bulking of data. This study illustrates the added value of using scATAC-seq rather than bulk ATAC-seq in evaluating both homogeneous and heterogeneous populations of cells. This paper provides a comprehensive guide on the benefits of using scATAC-seq data to study gene regulation.

## Introduction

The assay for transposase accessible chromatin using sequencing (ATAC-seq) can be used to profile open chromatin accessibility genome-wide^1^. The dynamic nature of chromatin accessibility reflects and correlates with the activity of genomic regulatory elements including enhancers, promoters and insulators. These elements account for a major proportion of the active non-coding genome in any cell type and contribute to the control of gene activity. They can initiate, activate, or suppress gene expression in a cell-type and developmental-stage-specific manner. Defining where and when these regulatory elements are active is crucial in understanding gene regulation and non-coding human genetics^2^.

The ATAC-seq assay has generated maps of regulatory elements in various cell types across many organisms. Nonetheless, these samples have often consisted of populations of cells, hence ATAC-seq describes the average chromatin accessibility of all cells in a sample. To characterize cell-type specific regulatory elements, many cell types or stages of differentiation in the lineage of these cell types need to be isolated prior to performing ATAC sequencing. Generating such collections with traditional ATAC-seq therefore requires intensive effort and resources. Additionally, isolation of particular cell types is not always feasible^3,4^.

The aggregated data from all cells within a sample cannot identify any inherent cellular and regulatory heterogeneity of differing cell types within the sample. The ability to isolate defined populations of cells from their heterogeneous tissue of origin is limited by the availability of methods for their identification and their isolation^5,6^. Recent technological and computational advances have made it possible to map chromatin accessibility at a single-cell level with single cell ATAC-seq (scATAC-seq), which has allowed for the simultaneous analysis of chromatin accessibility landscapes of heterogeneous mixtures of cells^7^. These outputs can be used to computationally cluster cells with similar chromatin accessibility profiles within a complex tissue type or a mixture of heterogeneous cell populations. This can lead to discovering of new cell populations, identifying rare cell types and defining new chromatin accessibility landscapes throughput processes such as differentiation and cellular activation. Aggregation of the data from these cell clusters (pseudo-bulking) can then be used to generate a genome-wide chromatin accessibility signal analogous to bulk ATAC-seq from a purified population^5,8,9^.

Resolving a heterogeneous population of cells in this way requires much less technical effort and cost than would be required with standard ATAC-seq. Nevertheless, scATAC-seq is a relatively novel technology and several questions need to be answered to understand its full capabilities^9,10^. First, we have addressed whether the chromatin accessibility profiles from bulk ATAC-seq and scATAC-seq differ in their structure. In this study, we compare the profiles derived from bulk ATAC-seq and scATAC-seq data from late erythroblast cells differentiated from CD34 cells from the same individual. We also used previously generated bulk ATAC-seq data from Natural Killer cells and compared this to publicly available scATAC-seq data from peripheral blood mononuclear cells (PBMCs) and see comparable results. Secondly, we have established the minimum number of cells required to annotate the genome of a heterogeneous cell cluster and have assessed the effect of cell number on data quality. This study therefore provides general guidance on the application and interpretability of single cell ATAC-seq from experiment to data analysis.

## Methods

### ATAC-seq data analysis for erythroblast and natural killer cells

**Fig. 1:**
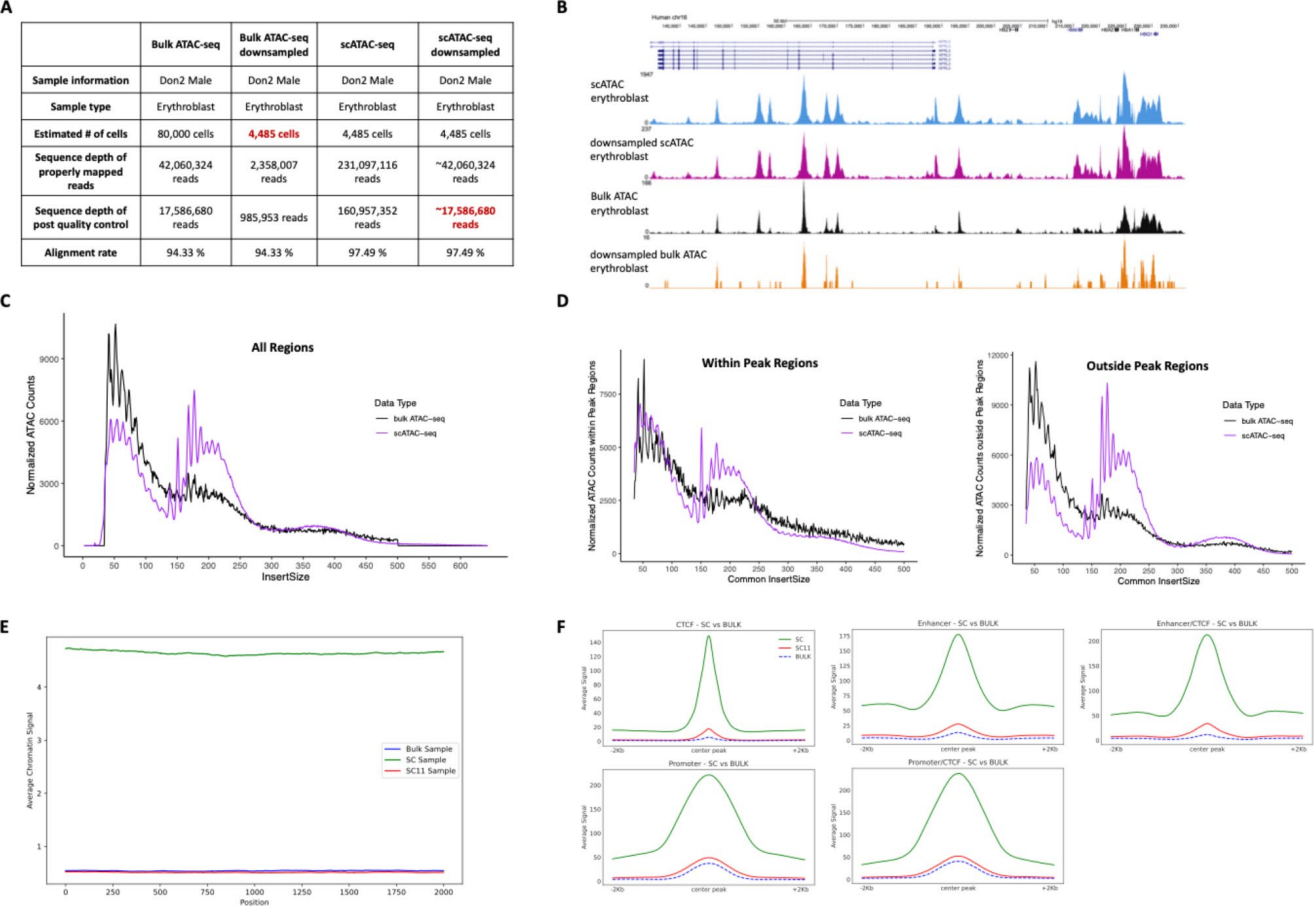
The scATAC-seq erythroblast data retains the same signal architecture with much fewer cells than bulk ATAC-seq erythroblast data. (**A**) Sequencing information for bulk ATAC-seq, scATAC-seq and downsampled versions of both. In the quality control step, firstly, only properly mapped pairs with MAPQ > 30 are extracted. Then, PCR duplicates were removed from those reads. (**B**) Downsampled scATAC-seq erythroblast data retains signal structure more accurately than bulk ATAC-seq. Comparison of data quality for the erythroblast dataset in scATAC-seq (blue track), downsampled scATAC-seq (purple track, downsampled from 160,957,352 reads to ~ 17,586,680 reads, ~ 11% of scATAC-seq reads), bulk ATAC-seq (black track) and downsampled bulk ATAC-seq (orange track, downsampled from 80,000 cells to 4,485 cells) at the alpha-globin locus. The distribution of DNA fragment sizes for the erythroblast dataset in bulk ATAC-seq (black) and scATAC-seq (purple) (**C**) shows a similar distribution trend with the difference in sequencing depth. The x-axis of distribution plots represents the fragment size of tagmented DNA, the y-axis shows a count frequency for those fragments. The decrease at around 150 bp (mono-nucleosomal) and 300 bp (di-nucleosomal) refers to the nucleosomal positioning. (**D**) The distribution of DNA fragment sizes within peaks (the left panel) and outside peaks (the right panel) in bulk ATAC-seq (black) and scATAC-seq (purple). (**E**) 50,000 regions from outside the common peak set were randomly selected and expanded by 2 kb on each side. The read count for these background regions is shown in blue (bulk ATAC-seq), green (scATAC-seq) and red lines (the downsampled scATAC-seq). (**F**) The average chromatin accessibility signal generated in the two assays over subclasses of active regions: CTCF, Promoter, Enhancer, Promoter/CTCF, Enhancer/CTCF.

Bulk ATAC-seq erythroblast data from a normal human donor was generated as part of a collaboration^11^. Bulk ATAC-seq data for Natural Killer (NK) cells was utilized in this study, previously generated in our lab from the same donor as the erythroblast dataset^12^. The ATAC-seq libraries were prepared and sequenced according to standard protocols to ensure high-quality chromatin accessibility profiling. Detailed information on the data generation process can be found in ^11^ and ^12^. The raw sequencing reads were aligned to the human genome hg19 or hg38 using Bowtie2 (‘-N 1’) (v2.4.2), only properly mapped read pairs were retained (42,060,324 reads for erythroblast) followed by removing low-quality reads (MAPQ < 30), unmapped reads, and PCR duplicates from the aligned data using SAMtools (v1.17). After the data quality filter step, 17,586,680 reads remained for erythroblast and 38,368,712 reads for NK cells. A coverage track was generated using deepTools BamCoverage (‘bamCoverage --binSize 1 –normalizeUsingRPKM’) (v3.5.2) to visualise bulk ATAC-seq erythroblast data on the UCSC genome browser (Fig. 1B). Finally, peak calling was performed via Lanceotron (lanceotron callPeaks), called peaks in both datasets were filtered by peak score (> 0.5), and blacklisted regions were discarded from the peak files.

### scATAC-seq data analysis for erythroblast

#### 10X analysis

The cellranger-atac count pipeline (10X Genomics Cell Ranger ATAC, v1.1.0) was used to analyse scATAC-seq erythroblast data. It aligned sequencing reads to the human genome hg19, detected peaks and generated a cell-by-peak matrix. 10X pipeline also generated a sorted and indexed BAM file containing all reads. The sequencing reads in the BAM files were filtered using SAMtools (v1.17) to keep only high-quality reads for downstream analysis. Only properly mapped read pairs were retained with the following flag ‘-f 3’ (231,097,116 reads) and all unmapped reads were filtered out with the following flag ‘-F 4’. Reads with mapping quality (MAPQ) score of 30 or higher were retained and duplicates were removed (samtools rmdup) (160,957,352 reads). For visualisation purposes, the coverage tracks were generated using deepTools (v3.5.2) with the following parameters: ‘bamCoverage --binSize 1 –normalizeUsingRPKM’. Peak calling was then performed on RPKM normalized bigwig file via LanceOtron (lanceotron callPeaks)^13^. Called peaks were then filtered by removing blacklist regions and retained peaks with an overall peak score greater than 0.5. Finally, data were visualised on the UCSC genome browser^14^ (Fig. 1B).

#### cisTopic analysis

After running the 10X count pipeline on scATAC-seq the erythroblast data, the cisTopic tool was used for downstream analysis. cisTopic leverages topic modelling, particularly Latent Dirichlet Allocation (LDA), to identify co-accessible chromatin regions (topics) across cells, which is more biologically meaningful than clustering cells or peaks separately^15^. The cisTopic object was created using the cell-by-peak matrix from the 10X results, followed by running the runWarpLDAModels function with the following parameters:

- α = 50,
- β = 0.1,
- iterations = 500,
- a number of topics = x, where x is in the range of 2 and 100 (2, 10, from 20 to 60, 1 by 1; from 70 to 100, 10 by 10).

With the selectModel function, the number of topics was determined as 48 after assessing the second derivative of the likelihood curve and perplexity. Then, for visualisation purposes, 2D t-Distributed Stochastic Neighbour Embedding (t-SNE) were calculated using the Rtsne R package (v0.17) (Fig. 3A).

### Gene ontology (GO) enrichment analysis

After performing cisTopic analysis on scATAC-seq erythroblast data, we detected two main clusters (Fig. 3A). To further understand the difference between these clusters, gene ontology (GO) enrichment analysis was performed on both peak sets (the small and large clusters in Fig. 3A). For GO enrichment analysis, enrichGO function from the clusterProfiler R package (v4.8.1) was used with default parameters for three orthogonal ontologies (biological process (BP), cellular component (CC) and molecular function (MF))^16^. For pathway enrichment analysis, enrichKEGG function from the same R package was used with default parameters. All enrichment results for two peak sets were merged in R using the tidyverse package (v2.0.0).

### Peripheral blood mononuclear cells (PBMCs) data analysis

#### 10X analysis

To reduce computation running time, a small PBMC multiome dataset containing approximately 3,000 cells (data: https://www.10xgenomics.com/datasets/pbmc-from-a-healthy-donor-granulocytes-removed-through-cell-sorting-3-k-1-standard-2-0-0) was used to demonstrate the required number of cells to form robust clusters. Therefore, the ATAC fragment information file and filtered feature barcode matrix, analysed by Cell Ranger ARC (10X Genomics, v2.0.0), were downloaded from the 10X Genomic website^17^.

#### ArchR analysis

After downloading the fragments file from the 10X Genomics for scATAC-seq PBMC, the ArchR package (v1.0.2) was used to form clusters^18^. Firstly, an arrow file was created with the following parameters:

- filterTSS = 4,
- filterFrags = 1000,
- addTileMat = TRUE,
- addGeneScoreMat = TRUE.

Then, the ArchR project was created after calculating doublet scores and filtering out doublets based on these scores (using the addDoubletScores function with default parameters). The workflow was explained below with default parameters used for each step.

1. The iterative LSI dimensionality reduction was implemented using the addIterativeLSI function on the tile matrix.
2. Uniform Manifold Approximation and Projection (UMAP) was added via the addUMAP function.
3. The plotEmbedding function was then used to visualise data in 2D space and cells were coloured by cluster information.
4. Finally, the addClusters function was used to form clusters (Fig. 5).

### Natural Killer (NK) cells from PBMC data analysis

Additionally, we utilized previously generated data with bulk ATAC-seq NK cells in the lab to make a robust comparison between the two methods. Since we did not have matching scATAC-seq NK cells from the same donor, we used publicly available 10X Genomics PBMC datasets (Table 1). To reach a similar number of cells as we had in scATAC-seq erythroblast (4,485 cells) data, we merged five different PBMC datasets. For those five datasets, their “filtered_peak_bc_matrix” files in h5 format were downloaded. These matrices were uploaded into the Azimuth ATAC app (https://app.azimuth.hubmapconsortium.org/app/human-pbmc-atac) for preprocessing and annotation analysis^19^.

**Table 1:** A list of five different 10X Genomics PBMC datasets and their sources.

10X Datasets	Source
10k PBMC using Next Gem	https://www.10xgenomics.com/datasets/10-k-peripheral-blood-mononuclear-cells-pbm-cs-from-a-healthy-donor-next-gem-v-1-1-1-1-standard-2-0-0
10k PBMC using Chromium Controller	https://www.10xgenomics.com/datasets/10-k-human-pbm-cs-atac-v-1-1-chromium-controller-1-1-standard-2-0-0
10k PBMC using Chromium X	https://www.10xgenomics.com/datasets/10-k-human-pbm-cs-atac-v-1-1-chromium-x-1-1-standard-2-0-0
10k PBMC using Chromium Controller v2	https://www.10xgenomics.com/datasets/10k-human-pbmcs-atac-v2-chromium-controller-2-standard
10k PBMC using Chromium X v2	https://www.10xgenomics.com/datasets/10k-human-pbmcs-atac-v2-chromium-x-2-standard

Cell barcode (CB) tag, which contains a unique identifier for each cell, related to NK cells (4,119 cells) were extracted from Azimuth ATAC annotation results (Supplementary Fig. 4A). 10X PBMC BAM files were first filtered as described for erythroblast scATAC-seq data analysis. And then further subsetted to extract NK cells using a modified version of the online script (https://divingintogeneticsandgenomics.com/post/split-a-10xscatac-bam-file-by-cluster/). The coverage tracks were generated using deepTools (v3.5.2) as explained before. Finally, peak calling was performed using LanceOtron (lanceotron callPeaks)^13^, followed by filtering called peaks by peak score (> 0.5), and removing blacklisted regions from the peak file.

### Peak annotation

#### Genomic coordinates-based peak annotation

**Fig. 2:**
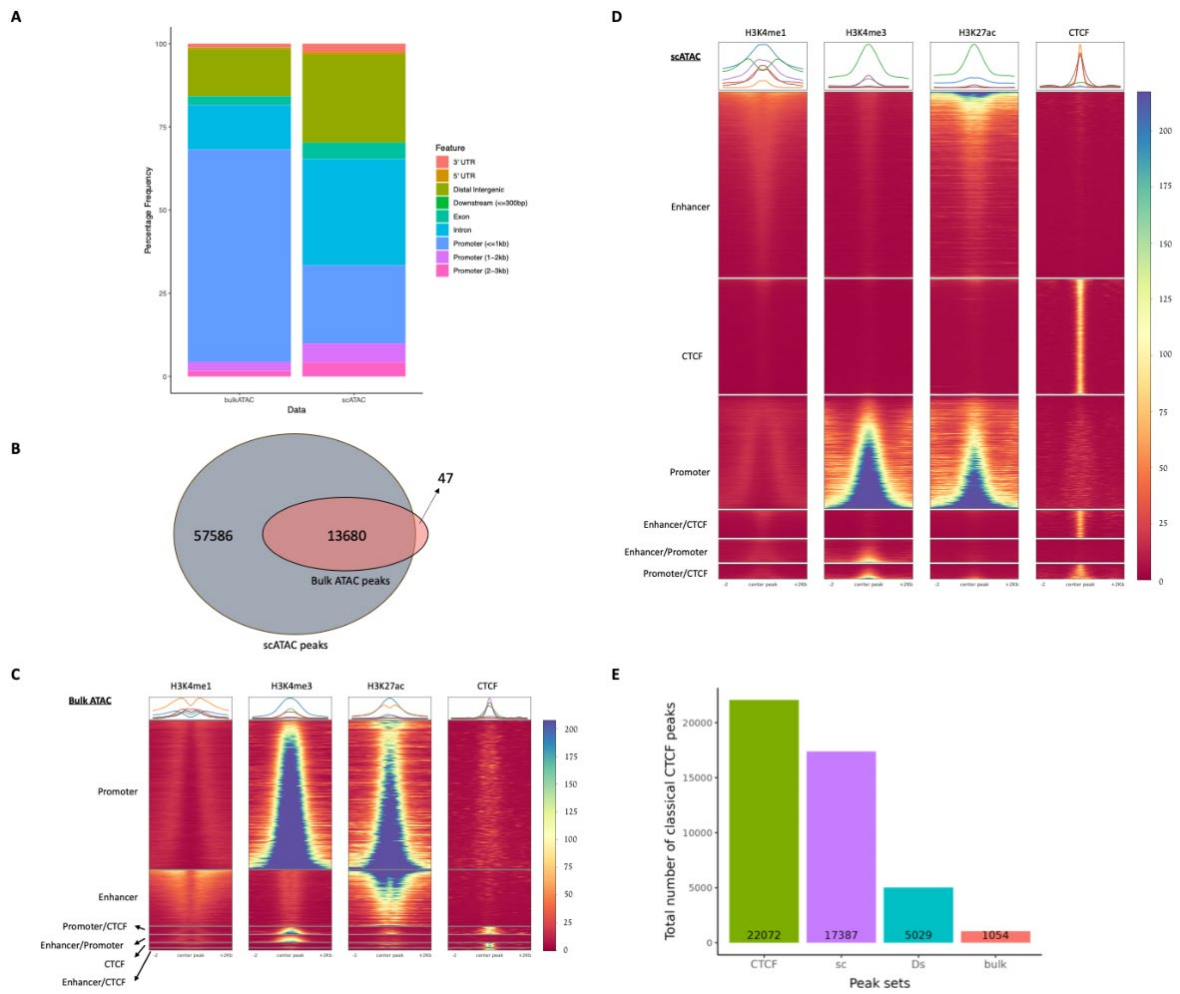
scATAC-seq identifies many more open chromatin regions than bulk ATAC-seq particularly, CTCF-bound-regions and potential primed-distal-elements. (**A**) Genomic location-based peak annotation for bulk ATAC-seq (left) and scATAC-seq peaks (right). scATAC-seq peaks provide a richer source of information on distal regulatory elements when compared to bulk ATAC-seq peaks. (**B**) The difference between bulk ATAC-seq and scATAC-seq peaks is shown as a Venn diagram representing the total number of peaks in each sample. (**C**) Peaks in bulk ATAC-seq were centred and expanded 2 kb from each side. Read count for those fixed regions was acquired from H3K4me1, H3K4me3, H3K27ac and CTCF. Then, peaks are categorised based on their epigenetic signatures. The peak annotation results for bulk ATAC-seq peaks by using publicly available ChIP-seq datasets show bulk ATAC-seq poorly identifies weak open chromatin regions such as CTCF sites. (**D**) Peaks in scATAC-seq were centred and expanded as 2 kb from each side. Read count for those fixed regions was acquired from, respectively, H3K4me1, H3K4me3, H3K27ac and CTCF. Then, peaks are categorised based on their on their epigenetic signatures. The peak annotation results for scATAC-seq peaks by using in-house ChIP-seq datasets show that scATAC-seq is more sensitive in the detection of CTCF sites. (**E**) The first bar plot represents the total number of classical CTCF peaks, the rest shows the total number of overlapping peaks between CTCF and open chromatin data (purple: scATAC-seq, blue: downsampled scATAC-seq (~ 11% of scATAC-seq reads) and red: bulk ATAC-seq). Related MLV^25^ session can be accessed through the link, https://mlv.molbiol.ox.ac.uk/projects/multi_locus_view/6274.

**Fig. 3:**
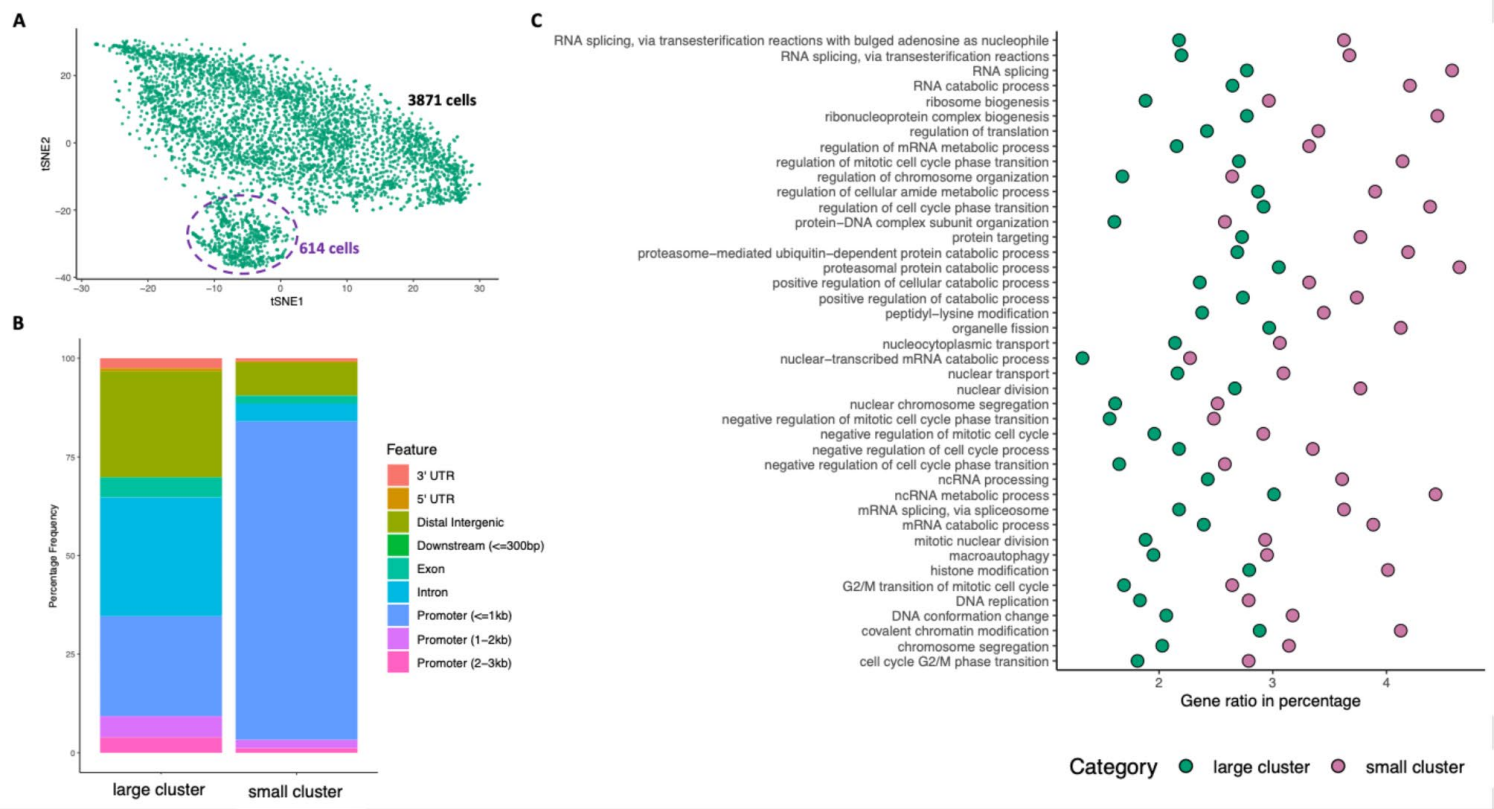
scATAC-seq can detect differences in chromatin accessibility in a presumed homogenous population. (**A**) Dimensionality reduction analysis for scATAC-seq of a population of erythroblasts is shown as a t-SNE projection. Each dot represents a single cell. Cells within the dotted purple circle demonstrate a different epigenomic landscape from other cells. (**B**) Genomic location-based peak annotation for the large cluster (top) and the small cluster (bottom). Peaks in the small cluster are identified as very close promoters, whereas the activity of distal regulatory elements seems to diminish. (**C**) Gene ontology enrichment result of the small cluster highlights regulation of the processes known to be involved in the ejection of the nucleus during red blood cell development. GO enrichment results of biological process (BP) in the small cluster (pink dots) and the large cluster (green dots) are shown as a dot plot. The x-axis shows the gene count ratio as a percentage, whereas the y-axis refers to enriched biological processes that are found by the algorithm after the false discovery rate (FDR) control.

The filtered peak files from bulk ATAC-seq and scATAC-seq were imported into R separately using the tidyverse R package (v2.0.0) as data frames and converted into GRanges objects with using the GenomicRanges R package (v1.52.1). annotatePeak function from the ChIPseeker R package (v1.36.0) was used to annotate peaks based on the UCSC genome annotation. The results from annotatePeak function were visualised using the ggplot2 R package (v3.5.1) with the following parameters: geom_bar(stat = “identity”) (Figs. 2A and 3B and Supplementary Fig. 4B).

### Peak annotation using publicly available ChIP-seq marks

Another way of annotating peaks is using specific ChIP-seq marks, such as H3K4me1, H3K4me3, and H3K27ac, to detect primed enhancer, promoter and general transcription activity^20^. The publicly available ChIP-seq datasets of these histone modifications, CTCF, and background input were used for this purpose^21^. For visualisation in the UCSC Genome Browser, coverage tracks were generated using deepTools BamCoverage (v3.5.2) for ChIP-seq BAM files. For both peak files, all peaks were extended by 2 kb from the center of the peak in each direction (in Python) to capture the ChIP-seq signal correctly. Coverage information was acquired from ChIP-seq BAM files for these extended peaks in both peak sets via bedtools multicov. Once read counts were obtained, the extended peak files were sorted by the difference between H3K4me1 and H3K4me3. For plotting, computeMatrix reference-point from deepTools (v3.5.2) was used to create matrices for bulk ATAC-seq and scATAC-seq data between ChIP-seq marks and peak files using the following parameters:

--referencePoint = center,
--beforeRegionStartLength = 2000,
--afterRegionStartLength = 2000,
--sortRegions = keep,
--skipZeros,
--numberOfProcessors = max.

**Fig. 4:**
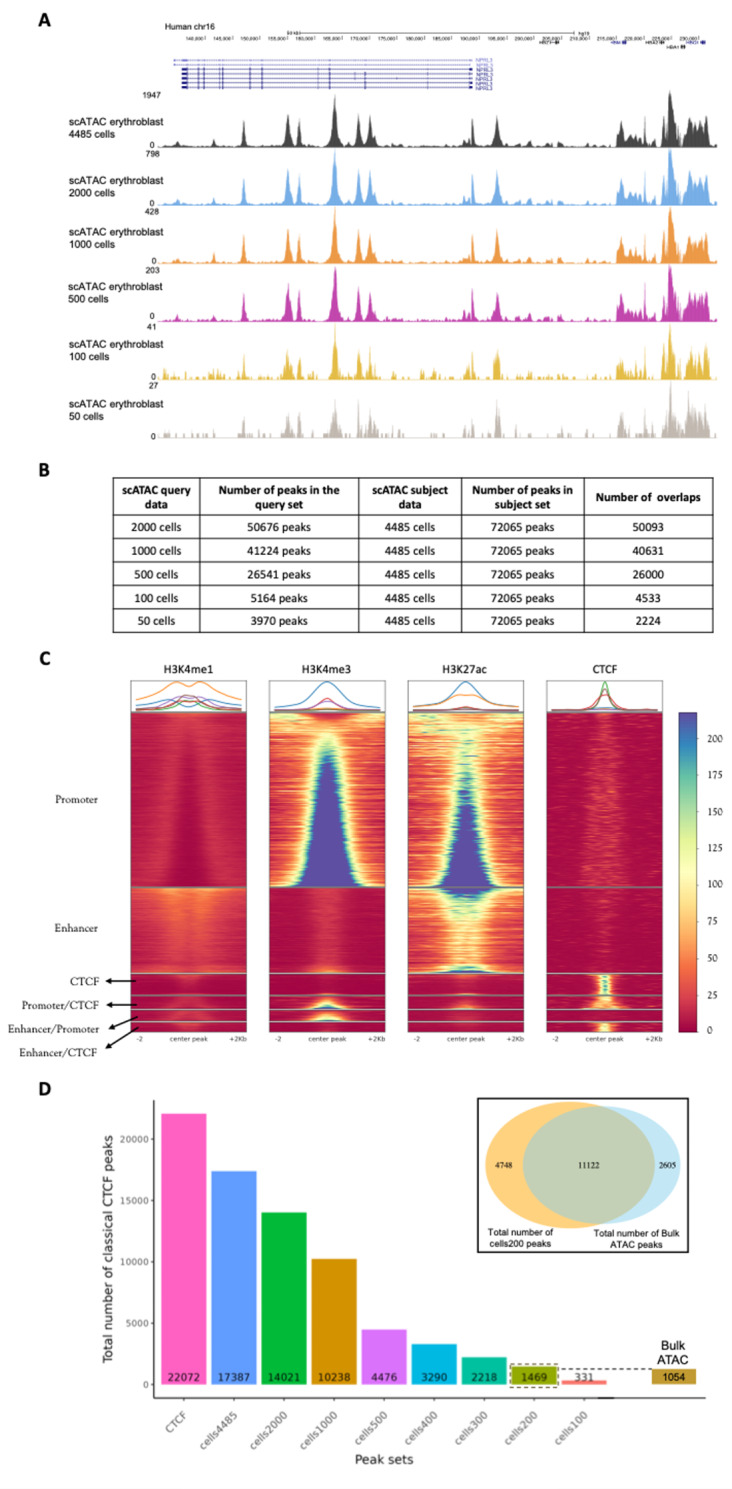
Data quality in scATAC-seq erythroblast data is high, with even 500 cells. (**A**) Comparison of data quality for scATAC-seq erythroblast data to cell number at the alpha-globin locus. The black track, 4,485 cells; the blue track, 2,000 cells; the orange track, 1,000 cells; the purple track, 500 cells; the yellow track, 100 cells; the grey track, 50 cells. (**B**) The total number of overlapping peaks between scATAC-seq erythroblast and its downsampled versions. (**C**) Peak annotation results for peaks from scATAC-seq erythroblast downsampled data containing 500 cells using the ChIP-seq datasets shows that as the number of cells reduces, the sensitivity of scATAC-seq data to identify CTCF sites diminished. Peaks in scATAC-seq data, containing 500 cells, were centred and expanded as 2 kb from each side. Read count for those fixed regions was acquired from H3K4me1, H3K4me3, H3K27ac and CTCF. Then, peaks are categorised based on their read coverage values. (**D**) The first bar plot represents the total number of classical CTCF peaks that were detected from ChIP-seq CTCF from the same individual, the subsequent bars show the total number of overlapping peaks between CTCF and scATAC-seq erythroblast downsampled versions.

Once matrices were generated, plotHeatmap from deepTools (v3.5.2) was performed to create a heatmap of each peak in each peak file as well as a line plot for average profile. Heatmaps and line plots were modified in Python using pandas (v2.1.4), NumPy (v1.24.3), matplotlib (v3.7.1) and seaborn (v0.12.2) packages. Then, peaks were categorised based on the read coverage from ChIP-seq data (Figs. 1E-F, 2C-D and 4C)^20^.

For supplementary Fig. 2, only peaks that are unique to scATAC-seq data were retained (57,586 peaks in Fig. 2B) and performed genome-wide heatmap using the same procedure explained before for the peak annotation using publicly available ChIP-seq marks (Supplementary Fig. 2).

For the NK cell population, publicly available ChIP-seq datasets (H3K4me1, H3K4me3 and CTCF) were downloaded from the ENCODE database^22–24^. The same analysis steps performed for the erythroblast data were also applied here (Supplementary Fig. 4C).

### In silico downsampling experiment

#### Homogenous data, erythroblast

scATAC-seq erythroblast data, which contains 4,485 cells, was downsampled to 2,000, 1,000, 500, 100, and 50 cells using a custom pipeline with Python packages, including pandas (v2.1.4), pysam (v0.21.0), tqdm (v4.65.0), os (v3.11.3), matplotlib (v3.7.1) and seaborn (v0.12.2).

The pipeline includes the following steps:

- Extracting CB tags from the analysis folder of the 10X count pipeline.
- Plotting read distribution for the tags.
- Downsampling the total number of cells to 2,000, 1,000, 500, 100 and 50 cells by randomly selecting CB tags in turn.
- Plotting read distribution for randomly selected tags.
- Subsetting the data by the total number of CB tags, and creating new BAM files.
- Sorting and indexing new BAM files.

After generating downsampled BAM files, the coverage tracks were generated using deepTools BamCoverage (v3.5.2) and uploaded onto the UCSC Genome Browser (Fig. 4A). Finally, peak calling was performed using LanceOtron (lanceotron callPeaks)^13^, followed by filtering called peaks by peak score (> 0.5), and removing blacklisted regions from the peak file.

#### ChIP-seq peak intersection with various downsampled scATAC-seq peaks

scATAC-seq erythroblast data, which contains 4,485 cells, was additionally downsampled to 400, 300, and 200 cells using the same script explained previously for in silico downsampling experiment to include more data. Finally, peak calling was performed using LanceOtron (lanceotron callPeaks) on both different versions of downsampled scATAC-seq sets (4485, 2000, 1000, 500, 400, 300, 200, 100 cells) and ChIP-seq promoter, enhancer and CTCF data^13^. bedtools intersect with the ‘-loj’ parameter was used to find overlapping regions between these scATAC-seq datasets and ChIP-seq datasets. This command outputs each peak in the ChIP-seq datasets along with overlapping regions from the scATAC-seq datasets. If there is no overlap, the output file keeps non-overlapping peak regions by filling them with placeholder values. The bar plots showing the number of overlaps between ChIP-seq datasets and scATAC-seq datasets were generated using the tidyverse R package (v2.0.0) (Fig. 4D, Supplementary Fig. 8A and B).

#### In silico dilution experiment

In silico experiment integrated two filtered scATAC-seq datasets: PBMC and erythroblast. Analysis of scATAC-seq erythroblasts showed that data, indeed, consists of two clusters: erythroblast and late erythroblast (Fig. 3). The large erythroblast cluster was extracted using CB tags in Python. Selected clusters and their total number of cells are as follows:

- Monocytes cluster: 842 cells,
- CD4 T cluster: 758 cells,
- CD8 naïve cluster: 288 cells,
- B cells cluster: 198 cells,
- CD8-other cluster: 129 cells,
- NK cells cluster: 106 cells,
- pDC cells cluster: 29 cells.

**Fig. 5:**
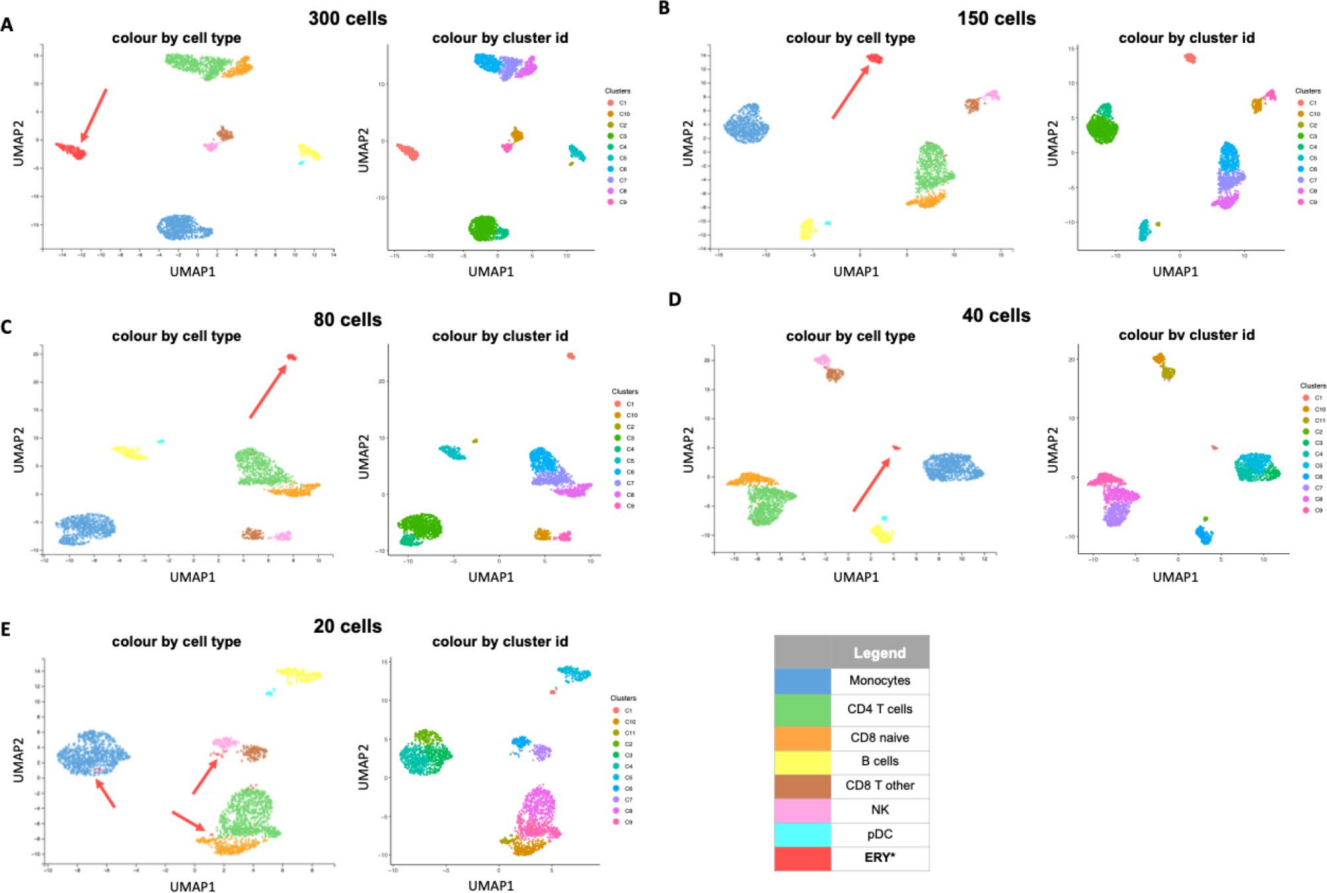
In silico experiment shows that clustering is still possible even with 40 cells. Each UMAP displays clustering results on different cell numbers for the erythroblast data as UMAP projections using ArchR. Each dot represents an individual cell and is coloured by cell type, as seen in the legend. (**A**) 300 cells; (**B**) 150 cells; (**C**) 80 cells; (**D**) 40 cells; and (**E**) 20 cells. The MLV session can be accessed through the link. https://mlv.molbiol.ox.ac.uk/projects/sc_atac_seq/5970.

A custom-made pipeline was used with the steps outlined as follows:

1. Extracting CB tags for homogenous erythroblast data (Fig. 5) and heterogenous PBMC data (B cells: Supplementary Fig. 9 and CD8 naïve cells: Supplementary Fig. 10).
2. Plotting read distribution for those tags.
3. Selecting CB tags randomly from the list in point 1, so the total number of diluted cells is 300 (198 for B and CD8 naïve cells), 150, 80, 40, and 20 cells.
4. Plotting read distribution for the randomly selected tags.
5. Subsetting the data by the total number of CB tags, and creating new BAM files.
6. Sorting and indexing new BAM files.
7. Merging subsetted homogenous erythroblast data with curated PBMC data.
8. Indexing merged BAM file.
9. Performing ArchR analysis on the merged data.
10. Merging different results from ArchR into one metadata and colouring UMAP by cell type and cluster ID (Fig. 5).
11. Saving metadata into a file in text format.

#### Assigning cell identity to clusters using multiome snATAC-seq and snRNA-seq

Azimuth app (https://azimuth.hubmapconsortium.org/) was used to analyse the gene expression matrix of snRNA of multiome dataset to get cell type annotation^19^. Azimuth performs cell identity assignment to clusters by leveraging the reference-based mapping method after normalising the count matrix. Annotation result provides three different levels of annotation depending on their granularity. Results from three different annotation methods were integrated together in R using the tidyverse R package (v2.0.0) and visualised in Multi-Locus View (MLV)^25^ (Fig. 5).

### Graphical analysis

#### Genome-wide chromatin accessibility patterns in bulk ATAC-seq and scATAC-seq erythroblast data

Only common peaks between bulk ATAC-seq and scATAC-seq data were retained (13,680 peaks in Fig. 2B) for this analysis. The common peak set was sorted by chromatin accessibility signal from scATAC-seq in descending order and this order was kept when generating the heatmap as explained previously for peak annotation using publicly available ChIP-seq marks (Supplementary Fig. 1A).

#### ATAC DNA fragment distribution

First of all, the distributions of DNA fragments for both bulk ATAC-seq and scATAC-seq data was plotted using all reads on chr16 with the R packages rtracklayer (v1.60.1) and Rsamtools (v2.16.0). The ggplot2 R package (v3.5.1) was used to plot the distribution using geom_line (Fig. 1C). Secondly, reads overlapping with common peak regions (13,680 peaks in Fig. 2B) were extracted from both bulk ATAC-seq and scATAC-seq data using SAMtools view (v1.17) with the ‘-L’ parameter. Next, these reads were excluded from both data to represent non-overlapping reads with the common peak file using bedtools intersect (v2.29.2) with the ‘-v’ parameter. The distributions of DNA fragments within and outside peak regions in both assays were plotted as explained before (Fig. 1D).

### Background estimation plot

Bedtools genomecov (v2.29.2) with the parameter ‘-bga’ is used to compute genome coverage by counting the number of reads that cover each base for bulk ATAC-seq and scATAC-seq erythroblast data. The base coverage files for bulk ATAC-seq and scATAC-seq are then used to calculate the percentage of total reads at each coverage level relative to the total reads in each data and visualise the results as percentages in the scatter plot (Supplementary Fig. 1B).

### Chromatin signal pattern outside the peak regions in erythroblast datasets

4 kb long 50,000 genomic regions were randomly selected from outside the union peak sets of bulk ATAC-seq and scATAC-seq. computeMatrix reference-point from deepTools (v3.5.2) was used to generate a signal matrix across the specified regions for comparison of signal patterns in the bulk ATAC, the scATAC-seq and the downsampled scATAC-seq data sets. Then, the matrix was imported into Python for visualization using pandas (v2.1.4), NumPy (v1.24.3), matplotlib (v3.7.1) and seaborn (v0.12.2) packages. The comparative line plots show chromatin signal across genomic positions (4 kb long 50,000 genomic regions outside the union peak sets) for three datasets (Fig. 1E).

## Results

### scATAC-seq improves data sensitivity compared to bulk ATAC-seq data

Although scATAC-seq data provides chromatin accessibility profiles at a single cell level, it is not clear whether it fully recapitulates the same signal structure as seen using bulk ATAC-seq data. In a previous study^11^, we generated data with both bulk ATAC-seq and scATAC-seq using the same input material allowing us to compare the two methods. Bulk ATAC and scATAC sequencing were performed on late erythroblasts derived from ex vivo differentiated CD34 + haematopoietic stem cells from a normal human donor. The input cell number for bulk ATAC-seq was around 80,000 cells, while for scATAC-seq was around 7,000 nuclei (4,485 cells recovered) and was sequenced to the recommended depth per cell for the 10X protocol (Fig. 1A)^11^. As the population was considered to be essentially homogeneous, the aggregate of all the single-cell data was used to compare bulk ATAC-seq and scATAC-seq from the same cells as genome-wide tracks of enrichment.

Visual assessment of the aggregated scATAC-seq data, at specific well-characterised active loci, such as the alpha-globin locus, showed very similar open chromatin patterns to the bulk ATAC-seq, although both data quality and depth appeared superior in scATAC-seq especially when the bulk signal was downsampled for number of input cells (Fig. 1A and B). Also, a genome-wide analysis of the common peaks called in both the bulk ATAC-seq and scATAC-seq showed them to be quantitatively similar in their intrinsic peak strength. Therefore, sorting by the signal strength of scATAC-seq from high to low, also sorts the bulk peaks from high to low signal (Supplementary Fig. 1A). These results showed that technology difference did not change the overall signal architecture, pattern of open chromatin sites or even relative signal strength of the peaks with the largest peaks in scATAC-seq also being the largest peaks in the bulk (Supplementary Fig. 1A). This, therefore, showed that aggregated scATAC-seq data can be used in an analogous manner to bulk ATAC-seq, but also suggested that scATAC-seq data from a substantial smaller number of cells could generate equivalent or even superior data to bulk ATAC-seq from a much larger number of cells.

Next, we tested if differences in the products of the transposition reaction in each technology might be different and so explain the differences in performance. The fragment sizes generated by each technology both show the same evident di-nucleosomal, mono-nucleosomal and sub-nucleosomal fragment distribution pattern in both bulk ATAC-seq (black line) and scATAC-seq (purple line) (Fig. 1C). While this shows that the Tn5 tagments chromatin into nucleosomal and subnucleosomal sized fragments in both technologies, an enrichment for both sub-nucleosomal and mono-nucleosomal fragments was evident within peak regions in scATAC-seq (Fig. 1D). In contrast, outside of peak regions in scATAC-seq was seen to be depleted for sub-nucleosomal but enriched for mono-nucleosomal fragments (Fig. 1D). Consequently, the background signal in bulk ATAC-seq is enriched for sub-nucleosomal fragments and depleted for nucleosomal fragments, relative to scATAC-seq. One potential reason for the apparent increase in signal in scATAC-seq could be that the bulk ATAC-seq protocol generates higher background due to its different tagmentation behaviour, which must be sequenced through to detect the signal. We, therefore, randomly selected 50,000 genomic regions (4 kb long) from outside the peak set and piled up the bulk, the full scATAC-seq and scATAC-seq signals subsampled to the same read depth as the bulk, over these inactive regions (Fig. 1E). While the background in the highly sequenced scATAC-seq dataset (green line) obviously appears much higher than in the bulk ATAC-seq (blue line), when the scATAC-seq is downsampled to the same sequence depth as the bulk sample (red line) levels of background appear comparable. Therefore, it appears that the increased sensitivity of scATAC-seq is not due to decreased levels of background generated in the scATAC-seq protocol. We next assessed the relative levels of signal generated in the two assays over active regions subclassified by types of active regions (‘CTCF’, ‘Promoter’, ‘Enhancer’, ‘Promoter/CTCF’, ‘Enhancer/CTCF’) (Fig. 1F). To perform this analysis additional erythroblast ChIP-seq datasets were used to determine regulatory element classes: H3K4me1, primed enhancer marker; H3K4me3, promoter marker; H3K27ac, active chromatin marker; and CTCF, boundary elements. With these data, both bulk ATAC-seq and scATAC-seq open chromatin peaks were annotated as to whether they were likely enhancers, promoters or CTCF sites. In each peak category, the downsampled scATAC-seq showed increased signal over each class of active element which was most evident over enhancers elements and CTCF bound regions. Using the scATAC-seq data at the recommended sequence depth (green line in Fig. 1F) showed an extremely large increase in signal-to-background. Considering this data was generated from only 4,485 cells (compared to 80,000 for bulk ATAC-seq), it showed that the scATAC-seq protocol generates extremely complex and information-rich ATAC libraries (Supplementary Fig. 1B). These high levels of library complexity, combined with increased signal over activity elements, therefore likely leads to the high levels of sensitivity per cell seen in scATAC-seq relative to bulk ATAC-seq.

This increased signal-to-noise within the scATAC-seq has the potential to increase the sensitivity of the scATAC-seq for the detection of open chromatin sites. Therefore, we compared the peak called output of the two data types and the number and types of active regions detected were compared. To do this, we applied the Lanceotron^13,26^ peak calling method to both data sets (total aggregated scATAC-seq and bulk ATAC-seq without downsampling). Both bulk ATAC-seq and scATAC-seq peaks were annotated based on genomic locations to determine if certain classes of elements, including promoters and distal regulatory elements, were detected differentially by the two technologies (Fig. 2). The most common class detected in the bulk ATAC-seq peaks are promoters (with a transcription start site (TSS) distance is less than 1 kb), while in scATAC-seq there is a large increase in distal intergenic and intronic peaks (Fig. 2A). Secondly, from a total of 71,313 peaks called, 57,586 peaks are only detected by scATAC-seq data, 47 peaks are unique to bulk ATAC-seq data and 13,680 overlap between both data sets (Fig. 2B). Therefore, almost every bulk ATAC-seq peak is also found in the scATAC-seq set. This supports the idea that single-cell ATAC-seq may simply be more sensitive than bulk ATAC-seq on a per cell basis, while the enrichment for promoter distal peaks would suggest that it is more effective in detecting enhancer and/or CTCF sites than bulk ATAC-seq.

Using our ChIP-seq based annotation showed that only a very small proportion of called peaks in bulk ATAC-seq are classified as CTCF sites, with the majority of peaks epigenetically marked as either enhancers or promoter classes (Fig. 2C). However, the same analysis in scATAC-seq peaks shows a very large increase in the detection of CTCF sites and, to a lesser degree, weakly marked active enhancer-like sites, suggestive of primed enhancers (Fig. 2D). This shows that bulk ATAC-seq has a much lower sensitivity than scATAC-seq for the detection of weak open chromatin sites, such as CTCF sites and weaker active enhancer-like elements (Supplementary Fig. 2). A very small number (*N* = 47) of enhancer and promoter peaks are unique to bulk ATAC-seq data. Manual inspection of these peaks showed them mostly to be due to minor variations in the peak callers’ behaviour (Supplementary Fig. 3) on the two datasets.

To quantify more objectively the difference in sensitivity between bulk ATAC-seq and scATAC-seq, we used annotated CTCF bound regions derived from the ChIP-seq analysis for these cells, devoid of overlapping enhancer or promoter chromatin marks. These sites represent the classical CTCF-bound sites that are not associated with chromatin marks and are typically associated with weak open chromatin peaks, making them more challenging to detect in ATAC-seq data. Of the 22,072 such classical CTCF sites, 17,387 (78.77%) were detectable by calling peaks from the aggregated scATAC-seq signal from all 4,485 cells. In comparison, only 1,054 (4.77%) of these sites were detectable when peak calling the bulk ATAC-seq sample derived from ~ 80,000 cells. This increased sensitivity is still seen after subsampling the scATAC-seq data to a comparable read depth with the bulk data, which detected 5,029 of the 22,072 CTCF sites (22.78%) (Fig. 2E), in agreement with the increased signal seen over CTCF sites in scATAC-seq (Fig. 1F).

In summary, the analysis of matched bulk ATAC-seq and scATAC-seq showed that, after pseudo-bulking, scATAC-seq data contained similar data but of superior sensitivity per cell of input than bulk ATAC-seq. This is important as it means it can therefore be used similarly to bulk ATAC-seq to annotate regulatory elements but at greater sensitivity at much lower cell numbers. This, therefore, opens the potential to use scATAC-seq data to annotate the epigenetic landscapes of multiple cell types simultaneously at high sensitivity in heterogeneous cell populations.

To confirm the generality of this conclusion, we made use of the publicly available scATAC-seq dataset from peripheral blood mononuclear cells and utilized previously generated bulk ATAC-seq data from Natural Killer cells (NK), which have a comparable number of cells to the large cluster in our erythroid dataset after merging (Supplementary Fig. 4A). This shows that a cluster of approximately four thousand cells produces a larger number of peak calls (58,753 vs. 39,778) for scATAC-seq compared to bulk ATAC-seq generated from 80,000 cells, with increased detection of CTCF bound sites (Supplementary Fig. 4B and C). However, due to the reuse of public data the bulk ATAC-seq and scATAC-seq are from different donors which likely explains the decreased overlap in the common peaks (39,778 peaks in total bulk ATAC-seq with 38,465 common between bulk ATAC-seq and scATAC-seq).

### scATAC-seq can detect differences in a presumed homogenous population

Identifying clusters of similar cells within complex heterogeneous populations is an essential but computationally intricate step in scATAC-seq analysis. Sparse data from each cell can be clustered to reveal distinguishable cell types and their epigenetic profiles. Bulk ATAC-seq of purified primary cells often assumes that they represent a homogenous population. scATAC-seq can detect differences in such “homogenous” populations. This can be seen in the dimensionality reduction and clustering analysis performed by Truch et al.^11^. The cells derived from the ex vivo differentiation of CD34+ stem cells have been considered a homogenous population of late erythroblasts, where the only expected differences would be related to cells in different stages of the cell cycle. However, the data analysis for this cultured sample clearly showed two distinct clusters of cells (Fig. 3A). To determine the reason for this, these two clusters were analysed further as individual cell types.

After applying peak calling on each cluster, peak sets were annotated based on where those peaks lie within the genome. This analysis showed a profound loss of peaks lying distal to TSSs in the smaller cluster (Fig. 3B). Gene ontology (GO) and pathway enrichment analysis using Kyoto Encyclopedia of Genes and Genomes (KEGG)^16^ was performed to understand the biological reason why the distribution of peaks in the small cluster might have changed drastically (Fig. 3C and Supplementary Fig. 5). The analysis showed a strong up-regulation of processes such as autophagy, macroautophagy, catabolism and chromatin modification (Fig. 3C and for full pathway analysis see Supplementary Fig. 5). These are all key processes in pyknosis that precedes the expulsion of the nucleus and mitochondria during red cell maturation^27^. Additionally, open chromatin profiles have previously been generated for the preceding stages of erythroid differentiation which would be the only other likely contaminating population in erythroid differentiation, and these do not match the profile of the small cluster even though it retains erythroid specific signals such as the enhancer regions of the alpha globin cluster (Supplementary Figs. 6 and 7). Therefore, this strongly suggests that this cluster represents cells derived from the larger cluster and represents a later erythroblast stage of differentiation, immediately prior to enucleation^28^.

This analysis showed that scATAC-seq not only recapitulates the same signal architecture as bulk ATAC-seq but also reveals cellular heterogeneity among and within presumed homogenous populations and allows for their separate analysis after pseudo-bulking of the detected clusters. The obvious difference in cell number between the large and small clusters highlights another important question in scATAC-seq: how many cells are enough to generate interpretable data equivalent to current bulk data?

### How many cells are required for an accurate genome annotation after pseudo-bulking?

To evaluate how many cells are required to obtain meaningful chromatin signatures after pseudo-bulking the scATAC-seq data (late erythroblast) was downsampled from 4,485 cells to 2000, 1000, 500, 100 and 50 cells. A visual assessment shows that data quality is relatively robust, down to 500 cells (Fig. 4A). At 100 cells, although the active regions are clearly discernible, the structure of the data is less well defined. While the extraction of useful data is still possible, it is more computationally challenging, and large losses in sensitivity are likely. To assess this globally, Fig. 4B shows the total number of peaks in each downsampled dataset and the number of peaks that overlap the original peaks obtained from analysing 4,485 cells (Fig. 4B). As the sensitivity diminishes, the number of overlaps between the downsampled data and the unsampled data clearly decreases. As it was seen that scATAC-seq and bulk ATAC-seq had differential sensitivity for detecting different classes of genomics elements, particularly CTCF sites, we assessed which classes were most sensitive to decreasing cell numbers.

Peaks from scATAC-seq erythroblast downsampled data containing 500 cells were categorised as a promoter, enhancer or CTCF using the ChIP-seq data to observe what type of regulatory element peaks were lost when sensitivity diminished. When the peak annotation results in Fig. 4C are compared with Fig. 2D, the majority of the peaks lost in the downsampled peak sets are CTCF sites. Thus, by reducing cell number and therefore, sensitivity of scATAC-seq data, regulatory elements like CTCF sites with weaker open chromatin signatures are more likely to be lost.

It would therefore be of great use for the practical application of scATAC-seq data to know approximately at what number of cells would the quality be equivalent to standard bulk ATAC-seq. Therefore, we again used CTCF-bound elements as an internal reference and progressively subsampled the scATAC-seq data until it was approximately as sensitive as the bulk data to the detection of these weak elements. We found that the aggregated scATAC-seq data from 200 cells detected a very analogous number of CTCF peaks to the bulk data (1,469 vs. 1,054 Fig. 4D). We also performed the same analysis for peaks annotated by chromatin marks as either enhancer and promoters and also showed approximately 200 cells gave a similar sensitivity to the bulk data (Supplementary Fig. 8). This suggests by these metrics that a cluster of around 200 cells in scATAC-seq would be of comparable sensitivity to standard bulk ATAC-seq.

### How many cells are required to form clusters robustly?

In the previous analysis, downsampling the total aggregated data was used to analyse the effect of cell number on data quality. However, in practice, we would analyse data from specific clusters identified within the data. It is therefore important to know not only that a cluster of a given number of cells will yield usable data, but also that a cluster of this size can be robustly identified during the dimensionality reduction and clustering steps to allow its subsequent aggregation and analysis. Therefore, the effect of the total number of cells on the formation of distinct clusters within the data was investigated to observe what diminished cell count accurate clustering would be impacted.

To do this, we chose to spike in data from cells of a highly defined cell population erythroblasts) into data from a highly heterogeneous population (PBMCs *N* = 3,000 cells) at different levels. Both data sets were derived from the 10X platform, and erythroblasts do not tend to exist in PBMC isolates. The aim was to determine the lowest number of erythroblast cells still capable of forming a distinct and identifiable cluster in the context of a highly complex sample. We chose to extract cell data from the large cluster shown in Fig. 3A to represent a homogenous erythroblast population and then spiked in 300, 150, 80, 40 or 20 cells into the PBMC data. Each spike-in dataset then went through the same analytical process with the same parameters using ArchR^18^. In this analysis, the spiked in erythroid cells are easily identified from the PBMC by tracking their unique cell IDs (CB tags). Clusters in the PBMCs were annotated using the gene expression data component of the multiome using a publicly available CITE-seq based reference database of PBMCs within the Azimuth platform^19^.

The scATAC-seq component of these multiome datasets was analysed independently using ArchR^18^, and then clusters were annotated via Azimuth^19^. Annotated monocyte, B cells, CD4 T cells, CD8 naive, CD8 T other, NK and pDC clusters were isolated to form distinct homogeneous clusters to avoid ambiguity in the identity of cells within each cluster. Since nucleated erythroid cells have very distinct epigenetic landscapes from these immune cell types, we hypothesised that they would form a unique cluster from the pre-existing cells.

Figure 5 clearly shows that the added erythroblast cells indeed formed a distinct cluster and were highly robust in terms of cell number. With 300, 150, 80 and 40 cells, the erythroblast cells were clustered together and distinct from all other cell types. However, with 20 erythroblast cells, the clustering algorithm failed to cluster them separately, with the algorithm instead assigning them to multiple clusters (Monocytes, NK cells, CD4 T cells and CD8 naive cells clusters).

Since erythroblast has very distinct cell populations compared to cell types in the immune niche, we also performed the same in silico experiment using the same procedure and parameters on B cells (Supplementary Fig. 9) and CD8 naïve cells (Supplementary Fig. 10) to test if the clustering was similarly robust when using cells which have more similar epigenetic landscape to PBMC cells used as a background. We therefore spiked in 198, 150, 80, 40 or 20 B and CD8 naïve cells into the PBMC data. Similar to the erythroid cell cluster used in the previous analysis these in silico dilution experiments showed that distinct clusters can be identified robustly with even 40 cells showing that these results are generalisable to different cell populations with epigenetic landscapes less distinct from the cellular background than erythroid cells. This is important as 40 cells is much lower than the number of cells we determined necessary to annotate a cell cluster’s epigenome via pseudo-bulking; therefore, it would suggest that if a cell cluster has sufficient cell numbers to form an informative ATAC track, then we would generally also have sufficient cell numbers to be identified as a coherent cluster.

## Discussion

Since bulk ATAC-seq only provides an average measurement of chromatin accessibility profiles from a population of cells, the emergence of single-cell ATAC-seq technology has enabled us to accurately map specific groups of cells within relatively small populations^29^. Importantly, this study shows that results obtained by pseudo-bulking single cell data have the same signal architecture as bulk ATAC-seq. Additionally, scATAC-seq provides better sensitivity for the detection of weak open chromatin sites such as those associated with CTCF-bound regions and primed enhancers. In combination with the new and effective approaches to cluster cells with the same chromatin accessibility profiles, scATAC-seq produces highly detailed and cell-type specific regulatory maps from heterogeneous mixtures of cells.

Our comparison of the fragment distribution showed that the common tagmentation step in both technologies produces evident nucleosomal and sub-nucleosomal profiles both within and outside of open chromatin peaks, although the degree of nucleosomal and sub-nucleosomal contributions differ. However, this still results in very similar relative peak strengths within each assay such that the order of largest to smallest peaks remains the same in both assays (Supplementary Fig. 1A). This is obviously critical for the use of aggregated scATAC-seq data in place of bulk ATAC-seq. However, our analysis shows that while the degree of general genomic background is relatively similar in both bulk and scATAC technologies, there is increased signal-to-noise over active regions, in particular CTCF elements and enhancers (Fig. 1E and F). The underlying reasons for this are not fully understood. It may be due to the difference in the tagmentation profiles within active regions. This shows an elevated contribution from sub-nucleosomal fragments in scATAC-seq which may represent protection by transcription factors bound to these regions. We also see an elevated contribution from mono-nucleosomal fragments, which are potentially derived from flanking modified nucleosomes (Fig. 1D).

Another important finding of this study is that the scATAC-seq assay does not require large numbers of cells to produce informative and useful regulatory data. This study shows that a scATAC-seq cluster with ~ 200 cells provide data quality equivalent to bulk ATAC-seq data. While data sensitivity decreases with cell number, surprisingly 100 cells from scATAC-seq data still produce interpretable data. Importantly, current clustering approaches can effectively identify clusters with fewer cells than this (~ 40 cells), so this step does not practically limit its use in rare subpopulations in heterogeneous mixtures.

## Conclusions

The work presented here shows that scATAC-seq, rather than being sparse, is rich in epigenetic information at a per cell level. This emphasises the potential of these technologies for both the simultaneous discovery of rare but biologically important cell types, such as stem and multipotent progenitors, and the use of their regulatory landscapes to better understand their biology and non-coding genetics Overall, this study comprehensively compares bulk ATAC-seq and scATAC-seq on the same material for data quality and resolution, generating chromatin accessibility profiles, and discernment of cell populations. It provides more detailed insight into the usage of scATAC-seq data.

## Electronic supplementary material

Below is the link to the electronic supplementary material.

Supplementary Material 1

## Data Availability

The data used in this study is listed below. bulk ATAC-seq on erythroblast, bulk ATAC-seq on NK cells, scATAC-seq on erythroblast, sc-multiome (snATAC & snRNA) sequencing on PBMC, ChIP-seq H3K4me1 on cells acquired at day 13 of erythropoiesis differentiation, ChIP-seq H3K4me3 on cells acquired at day 13 of erythropoiesis differentiation, ChIP-seq H3K27ac on cells acquired at day 13 of erythropoiesis differentiation, ChIP-seq CTCF on cells acquired at day 13 of erythropoiesis differentiation, ChIP-seq H3K4me1, H3K4me3 and CTCF for NK cells are from the ENCODE database 22–24.Erythroblast datasets were generated as part of the collaboration 11, and are available in the NCBI GEO repository, GSE193038 and GSE193035. Their generation process can be found in Truch et al. 11. For PBMC datasets, publicly available 10X datasets were downloaded from the 10X Genomic website 17. The ChIP-seq datasets used and analysed in this study are available in the NCBI GEO repository, GSE244929 and their generation process can be found in Georgiades et al. 21. Bulk ATAC-seq NK cells from the same donor as erythroblast datasets is available in the NCBI GEO repository, GSE125164 12. For ChIP-seq datasets for NK cells from ENCODE were utilized, including the following processed data versions: ENCODE4 v1.6.1 GRCh38 (ENCAN910QXX) for H3K4me1, ENCODE4 v1.5.1 GRCh38 (ENCAN474VGF) for H3K4me3, and ENCODE4 v1.8.0 GRCh38 (ENCAN761NZE) for CTCF 22–24.

## Abbreviations

ATAC-seq: assay for transposase-accessible chromatin with sequencing
BP: biological process
CB: cell barcode
CC: cellular component
FDR: false discovery rate
GO: gene ontology
KEGG: Kyoto encyclopaedia of genes and genomes
LDA: latent Dirichlet allocation
MAPQ: mapping quality
MF: molecular function
MLV: multi-locus viewer
NK: natural killer
PBMC: peripheral blood mononuclear cells
scATAC-seq: single cell assay for transposase-accessible chromatin with sequencing
snRNA-seq: single-nucleus RNA sequencing
t-SNE: t-distributed stochastic neighbour embedding
TSS: transcription start site
UMAP: uniform manifold approximation and projection
