# Supplementary material for: scATAC-seq generates more accurate and complete regulatory maps than bulk ATAC-seq
Source: Sci Rep. 2025 Jan 29;15:3665. doi: 10.1038/s41598-025-87351-7 (PMC11779887; doi:10.1038/s41598-025-87351-7)
Supplement: Supplementary file 1 — Supplementary Material 1 [file 41598_2025_87351_MOESM1_ESM.docx]

**Supplementary Figures - scATAC-seq generates more accurate and complete regulatory maps than bulk ATAC-seq.**

E. Ravza Gur^1,2^, Jim R. Hughes^1,2*^

^1^ MRC WIMM Centre for Computational Biology, MRC Weatherall Institute of Molecular Medicine, Radcliffe Department of Medicine, University of Oxford, Oxford, OX3 9DS, UK.

^2^ MRC Molecular Haematology Unit, MRC Weatherall Institute of Molecular Medicine, Radcliffe Department of Medicine, University of Oxford, Oxford, OX3 9DS, UK

*Corresponding author. E-mail: [jim.hughes@imm.ox.ac.uk](mailto:jim.hughes@imm.ox.ac.uk)

**Supplementary Figures**

**Supplementary Fig. 1: Overall chromatin accessibility pattern is consistent in both assays and scATAC-seq data has more functional signals.** **A** Peaks that are common in both methods (13,680 common peaks in Fig. 2B) were centred and expanded as 2kb from each side. Read count for these fixed regions was acquired from, respectively, scATAC-seq and bulk ATAC-seq and both tracks are sorted from largest to smallest based on the scATAC-seq values. **B** Shows how coverage is distributed across the sequenced genome for bulk ATAC-seq and scATAC-seq. The y-axis shows percentage of the genome at different coverage levels whereas the x-axis reflects coverage level (read count). This shows an expansion of signal-to-noise over the small percentage of genome which represents signal rather than background.

**Supplementary Fig. 2: The majority of peaks are unique to scATAC-seq are CTCF sites.** Peaks that are unique to scATAC-seq (57,586 unique peaks in Fig. 2B) were centred and expanded as 2kb from each side. Read count for these fixed regions was acquired from, respectively, H3K4me1, H3K4me3, H3K27ac and CTCF. Then, peaks are categorised based on their read coverage values.


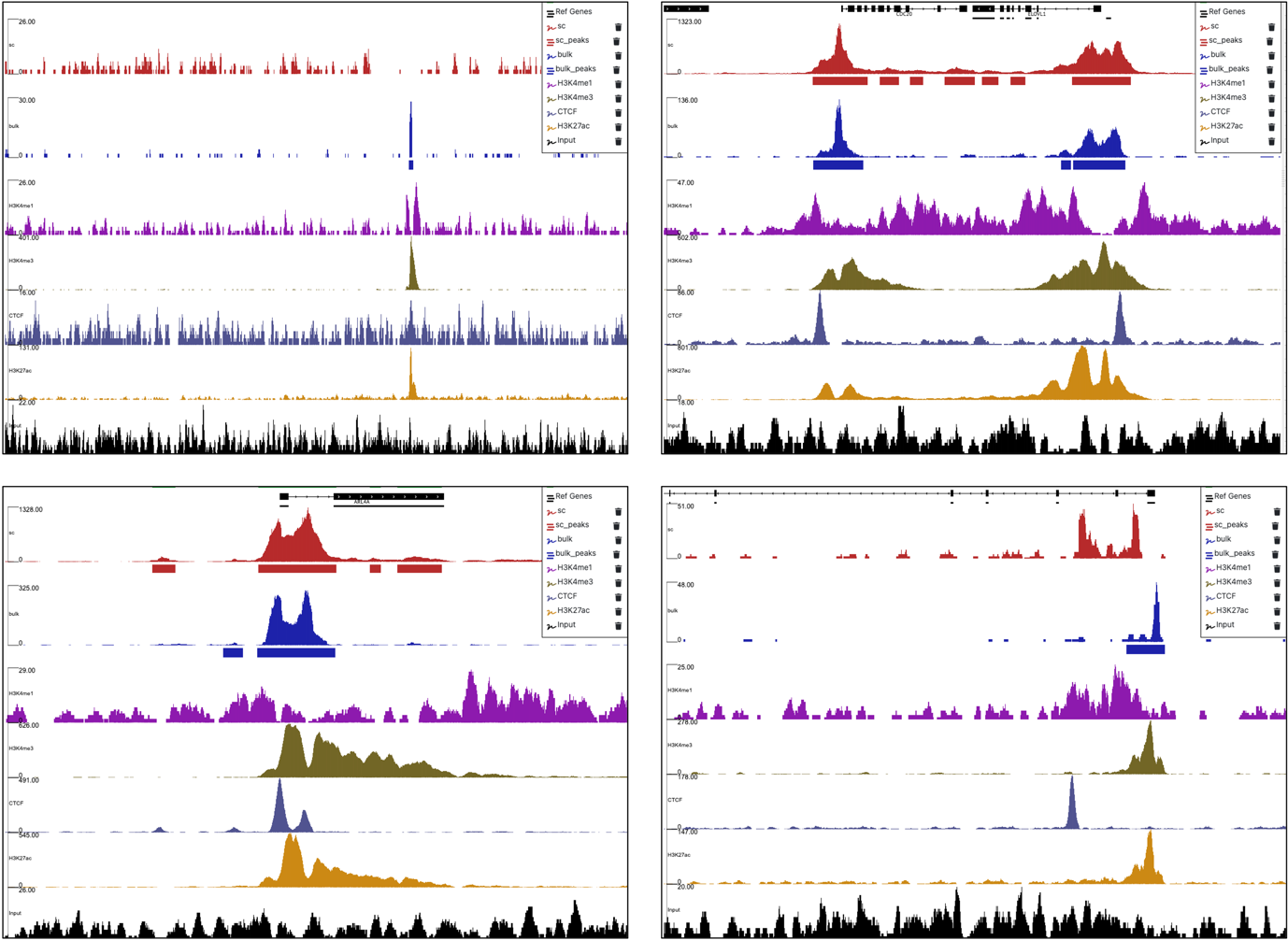


**Supplementary Fig. 3: Exclusive bulk ATAC-seq peaks (N=47) originated from the behaviour of peak callers to different backgrounds.** In each locus, the scATAC-seq erythroblast data is shown at the top of the figure in red, followed by its peak set in red blocks. The bulk ATAC-seq erythroblast data is beneath the scATAC-seq tracks in blue, followed by its peak set in blue blocks. The ChIP-seq markers are shown subsequently in order H3K4me1 (purple), H3K4me3 (light brown), CTCF (grey), H3K27ac (orange) and input (black).

**Supplementary Fig. 4: The scATAC-seq data for NK cells identifies more CTCF regions, even though it has a significantly smaller number of cells compared to the bulk ATAC-seq NK cell data. A** Sample information for bulk ATAC-seq and scATAC-seq NK cells. Peaks we called after retaining only properly unique mapped pairs with MAPQ > 30. **B** Genomic location-based peak annotation for bulk ATAC-seq (left) and scATAC-seq peaks (right). scATAC-seq peaks provided slightly more information about distal regulatory elements compared to bulk ATAC-seq peaks. **C** Peaks from bulk and single-cell data were centred and expanded by 2kb from each side. Read count for these fixed regions was obtained from H3K4me1, H3K4me3 and CTCF. scATAC-seq data identified more CTCF sites compared to bulk ATAC-seq data.

**Supplementary Fig. 5: Full gene ontology (GO) and pathway enrichment results in comparison to the small and large clusters demonstrate the regulation of processes associated with nuclear expulsion during red blood cell development.** GO enrichment results for biological process (BP) (A), cellular component (CC) (B), molecular function (MF) (C) and pathway enrichment (D) in the small cluster (pink dots) and the large cluster (green dots) are shown as a dot plot. The x-axis shows the gene count ratio as a percentage, whereas the y-axis refers to enriched biological processes that are found by the algorithm after the false discovery rate (FDR) control.

**Supplementary Fig. 6: The large cluster shows a similar chromatin accessibility pattern at the alpha-globin locus as Ludwig bulk ATAC-seq erythroid differentiation data** [30] **whereas the small cluster does not match this profile despite preserving some erythroid-specific signals.** The ATAC-seq signal respectively corresponds to the following cell types: MyP (myeloid progenitor cells), CFUE (colony-forming unit-erythroid cells), ProE1 and ProE2 (proerythroblasts), BasoE (basophilic erythroblasts), PolyE (polychromatic erythroblasts), and OrthoE (orthochromatic erythroblasts) from the study [30], and the small and large clusters from the scATAC-seq erythroblast data.

**Supplementary Fig. 7: The small cluster has demonstrated different chromatin accessibility landscapes compared to the large cluster suggesting that they are different cell types.** The chromatin signals at the alpha-globin **(A)**, the GATA2 **(B)**, the KLF1 **(C)** and the TAL1 **(D)** loci were shown for the large and small clusters in scATAC-seq erythroblast data.

**Supplementary Fig. 8: The scATAC-seq data with around 200 cells gives a similar sensitivity to the bulk ATAC-seq data.** The first bar plots in **(A)** and **(B)** respectively show the total number of promoter and enhancer peaks detected from ChIP-seq H3K4me3 and H3K4me1 data from the ENCODE database. The following Venn diagram in **(A)** demonstrate the number of peaks overlapping between H3K4me3 from ENCODE and downsampled scATAC-seq erythroblast data, while in **(B)** they demonstrate the number of peaks that overlap between H3K4me1 from ENCODE and downsampled scATAC-seq erythroblast data. **C** Comparison of data quality between scATAC-seq erythroblast data with 200 cells (black track) and bulk ATAC-seq erythroblast data (brown track) at the alpha-globin locus. The following Venn diagram shows the number of peaks overlapping between bulk ATAC-seq erythroblast and downsampled scATAC-seq erythroblast data.

**Supplementary Fig. 9: *In silico* experiment on B cell cluster shows that clustering is still possible even with 40 cells in a heterogenous population with closer epigenetic profiles, suggesting that this can be generalized.** Each UMAP displays clustering results on different cell numbers for erythroblast as UMAP projections using ArchR. Each dot represents an individual cell and is coloured by cell type, as seen in the legend. **A** 198 cells; **B** 150 cells; **C** 80 cells; **D** 40 cells; and **E** 20 cells.

**Supplementary Fig. 10: *In silico* experiment on CD8 naïve T cell cluster shows that clustering is still possible even with 40 cells in a heterogenous population with closer epigenetic profiles, suggesting that this can be generalized.** Each UMAP displays clustering results on different cell numbers for erythroblast as UMAP projections using ArchR. Each dot represents an individual cell and is coloured by cell type, as seen in the legend. **A** 198 cells; **B** 150 cells; **C** 80 cells; **D** 40 cells; and **E** 20 cells.
